# Combination therapies for cancer: challenges and opportunities

**DOI:** 10.1186/s12916-023-02852-4

**Published:** 2023-05-04

**Authors:** Zhijun Zhou, Barish H. Edil, Min Li

**Affiliations:** 1 Department of Medicine, The University of Oklahoma Health Sciences Center, 975 NE 10Th Street, BRC 1262A, Oklahoma City, OK 73104 USA; 2 Department of Surgery, The University of Oklahoma Health Sciences Center, Oklahoma City, OK 73104 USA

**Keywords:** Combinational therapy, Pancreatic cancer, Radical resection, Intestine auto-transplantation

## Abstract

**Background:**

Gastrointestinal cancers represent a major challenge to public health. Pancreatic cancer is the most lethal cancer among all gastrointestinal cancers. Most patients cannot meet the criteria of resection at diagnosis, indicating these patients will have dismal prognosis.

**Main text:**

Neoadjuvant chemotherapy helps some patients regain the opportunity of radical resection. An optimal regimen of chemotherapy is one that maximizes the anti-tumor efficacy while maintaining a relatively manageable safety profile. The development of surgical procedures further improves the outcomes of these patients.

**Conclusions:**

Combination therapies in a multidisciplinary manner that involves modified chemotherapy regimen, radical resection, and intestine auto-transplantation may provide the currently best possible care to patients with locally advanced pancreatic cancer.

## Background

Gastrointestinal cancers account for around 25.8% of newly diagnosed cancers annually. Among them, pancreatic cancer is the most lethal cancer, with a 5-year overall survival rate of only around 12%. There are more than 495,700 new cases of pancreatic cancer each year [1]. Unfortunately, over 80% of these patients lost the opportunity of surgery at diagnosis. Neoadjuvant chemotherapy helps some patients regain the opportunity of radical resection, the only way for possible cure. Numerous efforts have been made in hopes of increasing the efficacy while decreasing the side effects of chemotherapy. A widely-used chemotherapy agent is gemcitabine, which is usually combined with other drugs as an adjuvant or neoadjuvant treatment option. The first-line chemotherapy regimen is FOLFIRINOX consisting of fluorouracil, leucovorin, irinotecan, and oxaliplatin. Although this regimen achieved a relatively satisfying anti-tumor effect on metastatic pancreatic cancer, it would inevitably bring undesirable adverse effects, which reduced treatment efficacy [2]. Optimizing the dose of the FOLFIRINOX regimen is critical for the treatment efficacy.

## Combination therapy holds the promise

Liang and colleagues proposed a modified FOLFIRINOX regimen, consisting of 85% oxaliplatin, 75% irinotecan, and zero fluorouracil bolus [3]. This modification enhanced the tolerance against the FOLFIRINOX-related adverse events, without compromising the therapeutic efficacy. The median overall survival (OS) and progression-free survival (PFS) were 10.3 months and 7.0 months in metastatic pancreatic cancer patients with the treatment of the modified FOLFIRINOX regimen, comparable to 11.1 months and 6.4 months in patients receiving the traditional full-dose treatment [2]. For the elderly patients with advanced pancreatic cancer who potentially had a high risk of intolerability, this modified approach also demonstrated acceptable tolerance and high treatment efficacy [4]. In addition, racial difference between the Western and Eastern countries greatly influences chemotherapy tolerance and the related treatment standard, primarily due to the race-dependent physical differences [3]. For the Eastern populations, the dose modification would substantially decrease the incidence and severity of adverse effects. Dose modification therefore broadens the application of the FOLFIRINOX regimen, making it more feasible to the Asian populations. Notably, in comparison to the other dose modification approaches, Liang’s modification showed a remarkably reduced incidence of grade 3/4 adverse events and therefore substantially improved tolerance during the chemotherapy course [3]. This strategy has been accredited as a predominant chemotherapy regimen by many professionals at international congresses and consensus summits. This modified FOLFIRINOX regimen has been widely used in more than 20 pancreatic cancer centers globally.

The modified FOLFIRINOX regimen was also applied as a neoadjuvant treatment to borderline resectable pancreatic cancer (BRPC) and locally advanced pancreatic cancer (LAPC) for downstaging and creating surgical opportunity. According to the LAPACT trial that applied gemcitabine plus nab-paclitaxel to LAPC, the neoadjuvant induction therapy achieved a 15% conversion rate of radical resection [5]. Notably, neoadjuvant therapy with modified FOLFIRINOX in LAPC, studied by Liang in a large prospective cohort CISPD-4, showed a surgical conversion rate of 37.1% [6]. The median OS and PFS of LAPC patients that were qualified for surgical resection after treatment with modified FOLFIRINOX were 27.7 months and 19.3 months, respectively, which were similar to that in patients with resectable pancreatic cancer at diagnosis (30.0 months and 23.0 months).

The expanded surgical resections after neoadjuvant therapy are critical to the improvement of the radical resection rate in LAPC. For LAPC involving the pancreatic body/tail and the celiac trunk, radical resection can be achieved by the modified Appleby procedure (distal pancreatectomy with celiac artery resection). This group is one of the leading groups that focus on intestine auto-transplantation and liver cancer surgery [7, 8]. As for the LAPC at the pancreatic head, they proposed a novel surgical procedure combining the pancreaticoduodenectomy and intestine auto-transplantation following this modified FOLFIRINOX induction. This combination therapy improved the surgical conversion rate to 67.0%, which is much higher than traditional procedures [9]. Ablative radiotherapy and targeted therapy showed a promising anti-tumor effect in some patients with LAPC. Further studies are warranted to evaluate the role of this novel surgical procedure in combination with radiotherapy and targeted therapy in LAPC.

Liang’s integrated treatment strategy by combining modified FOLFIRINOX with sequential radical resection has established an excellent model of multimodal treatment against pancreatic cancer, and extended criteria of surgical resectability, which would benefit more pancreatic cancer patients at locally advanced stages. The success of this strategy implies that for pancreatic cancer patients, especially those who cannot meet the resectability criteria, the combination of modified chemotherapy and surgical intervention would provide an increased survival benefit. Furthermore, the interim analysis of a prospective phase 3 clinical trial (The CISPD3 trial) conducted by Liang’s group [10], comparing the therapeutic effect of modified FOLFIRINOX with modified FOLFIRINOX plus sintilimab (a PD-1 monoclonal antibody) in metastatic and recurrent pancreatic cancer, showed that the addition of immunotherapy improved the response rate of the modified FOLFIRINOX chemotherapy, indicating that the modified FOLFIRINOX chemotherapy may have a synergistic effect with immunotherapy in metastatic and recurrent pancreatic cancer in terms of response rate.

In summary, gastrointestinal cancers represent a major challenge to public health. Most patients cannot meet the criteria of resection at diagnosis, indicating these patients will have dismal prognosis. Neoadjuvant chemotherapy helps some patients regain the opportunity of radical resection. The development of surgical procedures further improves the outcomes of these patients. Combination therapies in a multidisciplinary manner that involves modified FOLFIRINOX regimen, radical resection plus intestine auto-transplantation, targeted therapy and immunotherapy may provide the best possible care to patients with LAPC.

## Conclusions

The modified FOLFIRINOX regimen is currently an optimal treatment option for pancreatic cancer patients. Combination of the modified FOLFIRINOX regimen, radical resection, and intestine auto-transplantation provides the currently best possible survival benefit for those who have otherwise lost the opportunity of surgery. The addition of immunotherapy, targeted therapy, and radiotherapy may further increase the efficacy of the combination therapy.

## Data Availability

Not applicable.

## Abbreviations

FOLFIRINOX: Folinic acid, fluorouracil, irinotecan and oxaliplatin
BRPC: Borderline resectable pancreatic cancer
LAPC: Locally advanced pancreatic cancer
OS: Overall survival
PFS: Progression-free survival
PD-1: Programmed cell death protein 1
